# A dataset of geocoded Medicaid office locations in the United States

**DOI:** 10.1016/j.dib.2024.110068

**Published:** 2024-01-15

**Authors:** Paul R. Shafer, Maxwell Palmer, Ahyoung Cho, Mara Lynch, Pierce Louis, Alexandra Skinner

**Affiliations:** a Department of Health Law, Policy, and Management, Boston University, 715 Albany Street Talbot 340 West, Boston, MA 02118, United States; b Department of Political Science, Boston University, United States; c School of Law, Boston University, United States; d Department of Environmental Health, Boston University, United States; e Department of Epidemiology, Brown University, United States

**Keywords:** Medicaid, Offices, Access, Enrollment, Caseworkers, Administrative burdens, Geocoding, Equity

## Abstract

Medicaid is the largest health insurance program in the United States, covering more than 86 million Americans as of early 2023, and is key for progress towards health equity. Although policy changes like Medicaid expansion have significantly expanded the number of people who are eligible for Medicaid, the administrative burdens of enrolling in and renewing coverage can be substantial. Although many applications are now submitted online, physical access to Medicaid offices still plays a critical role in understanding eligibility, getting help in applying, and navigating required documentation for both initial enrollment and redetermination of eligibility. However, as more government functions have moved online, in-person office locations and/or staff may have been cut to reduce costs, and gentrification has shifted where minoritized, marginalized, and/or low-income populations live, it is unclear if the key local connection point between residents and Medicaid has been maintained. To our knowledge, no single source of Medicaid office locations has been assembled and made available for research purposes. Our objective was to identify and geocode all public-facing Medicaid offices in the United States, which can then be paired with other spatial data (e.g., demographics, Medicaid participation, health care use, health outcomes) to explore policy-relevant research questions. We identified Medicaid office addresses in all 50 states and the District of Columbia by searching state government websites (e.g., Department of Health and Human Services or analogous state agency). Our corpus of Medicaid office addresses was then geocoded using the Census Geocoder with unresolved addresses investigated and/or manually geocoded using Google Maps. After deduplication (e.g., where multiple counties share a single office) and removal of mailing addresses (e.g., PO Boxes), our final dataset includes 3026 Medicaid office locations.

Specifications Table

**Table:** 

Subject	Social Sciences / Health
Specific subject area	Medicaid office addresses with geocoded latitude and longitude
Data format	Raw
Type of data	Table
Data collection	Three coders identified Medicaid office addresses in all 50 states and the District of Columbia by searching state government websites (e.g., Department of Health and Human Services or analogous state agency) during late 2021 and early 2022, which was then reviewed for accuracy by a fourth coder. Our corpus of Medicaid office addresses was then geocoded using the Census Geocoder with unresolved addresses investigated and/or manually geocoded using Google Maps. The corpus was updated in August through December 2023 following the end of the COVID-19 public health emergency by a fifth coder as several states closed and/or combined offices during the pandemic. After deduplication and removal of mailing addresses, our final dataset includes 3026 Medicaid office locations.
Data source location	Institution: Boston University, City/Town/Region: Boston, MA, Country: United States
Data accessibility	Repository name: Harvard Dataverse Data identification number: 10.7910/DVN/AVRHMI Direct URL to data: https://doi.org/10.7910/DVN/AVRHMI

## 1 Value of the data

• To our knowledge, no single source of public-facing Medicaid office locations providing enrollment support has been assembled and made publicly available in a suitable format for research, despite growing attention to the impact of administrative burdens on social safety net program participation and health.
• These data can be valuable to researchers in public administration, economics, health policy, and related fields for whom spatial data on public-facing Medicaid office locations can facilitate exploring previously unknown relationships, by pairing these data with other spatial data (e.g., demographics, Medicaid participation, health care use, health outcomes).
• Medicaid expansion has significantly expanded the number of people who are eligible for Medicaid and the creation of the health insurance marketplace under the Affordable Care Act created a very visible avenue through which people can learn that they are eligible.
• As more government functions have moved online, in-person office locations and/or staff may have been cut to reduce costs, and gentrification has shifted where minoritized, marginalized, and/or low-income populations live, it is unclear if the key local connection point between residents and Medicaid has been appropriately maintained.
• Although many applications are now submitted online, physical access to state, county, and tribal Medicaid offices still plays a critical role in understanding eligibility, getting help in applying, and navigating required documentation for both initial enrollment and redetermination of eligibility.

## 2 Background

Medicaid is the largest health insurance program in the United States, covering more than 86 million Americans as of early 2023 [1], and is key for progress towards health equity [2]. Although policy changes like Medicaid expansion have significantly expanded the number of people who are eligible for Medicaid, the administrative burdens of enrolling in and renewing coverage can be substantial and have known detrimental effects on health [3,4]. Although many applications are now submitted online, physical access to Medicaid offices still plays a critical role in understanding eligibility, getting help in applying, and navigating required documentation for both initial enrollment and redetermination of eligibility. However, as more government functions have moved online, in-person office locations and/or staff may have been cut to reduce costs, and gentrification has shifted where minoritized, marginalized, and/or low-income populations live, it is unclear if the key local connection point between residents and Medicaid has been maintained. To our knowledge, no single source of public-facing Medicaid office locations has been assembled and made publicly available for research purposes. These data can facilitate exploring previously unknown relationships related to administrative burdens, by pairing these data with other spatial data (e.g., demographics, Medicaid participation, health care use, health outcomes). For example, these data can facilitate research on the effects of access to Medicaid offices (e.g., distance, closures, public transit) on program participation, health care use, and/or health outcomes [[5], [6]]. They could also be paired with office locations for other social service programs (i.e., Social Security, Supplemental Assistance Nutrition Program, unemployment insurance) to understand whether and how much co-location or tighter clustering of agencies affects safety net program interactions, independent of eligibility and benefit generosity [7].

## 3 Data Description

The dataset is provided as a Stata file (offices_data.dta), SAS file (offices_data.v8xpt), and Excel file (.xlsx), each including identical data [8].

"state_fips": state FIPS code

"state_name": state name

"agency_name": agency name

"street1": street address

"street2": street address (continued)

"city": city

"state": 2-letter state abbreviation

"zip_code": ZIP code

"latitude": latitude

"longitude": longitude

## 4 Experimental Design, Materials and Methods

Our objective was to identify and geocode all public-facing Medicaid offices providing enrollment support in the United States for pairing with other spatial data (e.g., demographics, Medicaid participation, health care use, health outcomes) to investigate policy-relevant research questions. Three coders (AC, PL, ML) identified Medicaid office addresses in all 50 states and the District of Columbia by searching state government websites (e.g., Department of Health and Human Services or analogous state agency) during late 2021 and early 2022 for the appropriate Medicaid agency and its office locations, which were then reviewed for accuracy by a fourth coder (AS). The state government websites used to develop our corpus of Medicaid office locations are included as Table 1. We excluded locations that appear to only provide client services (e.g., behavioral health, home and community-based services for long-term care needs) to Medicaid enrollees or only house administrative functions, without providing face-to-face enrollment support. We followed links to county social service agencies for Medicaid office locations if included in the state listing as the source of locations for that county. We included closely neighboring government-operated Medicaid office locations (e.g., within a block or two on the same street) as a single location even if listed separately on the state government website. Our corpus of Medicaid office addresses was then geocoded using the Census Geocoder from the US Census Bureau (https://geocoding.geo.census.gov/geocoder/) with unresolved addresses investigated and/or manually geocoded using Google Maps. The corpus was updated in August through December 2023 following the end of the COVID-19 public health emergency by a fifth coder (PS) as several states closed and/or combined offices during the pandemic. After deduplication (e.g., where multiple counties share a single office) and removal of mailing addresses (e.g., PO Boxes), our final dataset includes 3026 Medicaid office locations.

**Table 1:** State government websites used to identify Medicaid office locations, August to December 2023.

State	URL
Alabama	https://medicaid.alabama.gov/content/10.0_Contact/10.1_Medicaid_Contacts/10.1.1_Medicaid_Locations.aspx
Alaska	https://health.alaska.gov/dpa/Pages/contacts.aspx
Arizona	https://azdes-community.my.salesforce-sites.com/EOL/; https://dbmefaapolicy.azdes.gov/index.html#page/FAA6/FAA_Local_Offices.html#
Arkansas	https://humanservices.arkansas.gov/contact-us/county-office-map/
California	https://www.dhcs.ca.gov/SERVICES/MEDI-CAL/Pages/CountyOffices.aspx
Colorado	https://cdhs.colorado.gov/contact-your-county; https://apps.colorado.gov/apps/maps/hcpf.map
Connecticut	https://portal.ct.gov/DSS/About-the-Department-of-Social-Services/Contact
Delaware	https://dhss.delaware.gov/dhss/dss/ofclocations.html
District of Columbia	https://dhcf.dc.gov/service/medicaid
Florida	https://www.myflfamilies.com/services/public-assistance/additional-resources-and-services/ess-storefronts-and-lobbies; https://access-web.dcf.state.fl.us/CPSLookup/search.aspx
Georgia	https://dfcs.georgia.gov/locations
Hawaii	https://medquest.hawaii.gov/en/resources/med-quest-offices.html
Idaho	https://healthandwelfare.idaho.gov/offices
Illinois	http://www.dhs.state.il.us/page.aspx?
Indiana	https://www.in.gov/fssa/dfr/ebt-hoosier-works-card/find-my-local-dfr-office/
Iowa	https://hhs.iowa.gov/location-by-county?gsl_feature_filter_list_value=iowa-healthcare&gsl_addressfield_locality=
Kansas	https://www.dcf.ks.gov/DCFContacts/Pages/default.aspx
Kentucky	https://prd.webapps.chfs.ky.gov/Office_Phone/index.aspx; https://www.chfs.ky.gov/agencies/dcbs/dsr/Documents/directoryofserviceregions.pdf
Louisiana	https://ldh.la.gov/index.cfm/directory/category/158; https://ldh.la.gov/page/262
Maine	https://gateway.maine.gov/dhhs-apps/office_finder/
Maryland	https://mydhrbenefits.dhr.state.md.us/dashboardClient/#/dssMap
Massachusetts	https://www.mass.gov/info-details/masshealth-enrollment-centers-mecs
Michigan	https://www.michigan.gov/healthymiplan/county-offices
Minnesota	https://mn.gov/dhs/people-we-serve/adults/health-care/health-care-programs/contact-us/county-tribal-offices.jsp
Mississippi	https://medicaid.ms.gov/about/office-locations/
Missouri	https://dss.mo.gov/dss_map/
Montana	https://dphhs.mt.gov/HCSD/OfficeofPublicAssistance
Nebraska	https://dhhs.ne.gov/Pages/Public-Assistance-Offices.aspx
Nevada	https://dwss.nv.gov/Contact/Welfare/; https://dhcfp.nv.gov/Contact/Contact_Home/
New Hampshire	https://www.dhhs.nh.gov/about-dhhs/locations-facilities#locations
New Jersey	https://www.nj.gov/humanservices/njsnap/home/cbss.shtml
New Mexico	https://www.hsd.state.nm.us/LookingForAssistance/Field_Offices_1/
New York	https://www.health.ny.gov/health_care/medicaid/ldss.htm; https://www.nyc.gov/site/hra/locations/medicaid-locations.page
North Carolina	https://www.ncdhhs.gov/localDSS
North Dakota	https://www.hhs.nd.gov/human-service/zones
Ohio	https://jfs.ohio.gov/about/local-agencies-directory
Oklahoma	https://oklahoma.gov/okdhs/contact-us/dhsofficelocations.html
Oregon	https://www.oregon.gov/odhs/Pages/office-finder.aspx?serviceid=22
Pennsylvania	https://www.dhs.pa.gov/Services/Assistance/Pages/CAO-Contact.aspx
Rhode Island	https://dhs.ri.gov/about-us/dhs-offices
South Carolina	https://www.scdhhs.gov/members/where-go-help
South Dakota	https://dss.sd.gov/findyourlocaloffice/
Tennessee	https://www.tn.gov/content/tn/humanservices/for-families/supplemental-nutrition-assistance-program-snap/office-locator-family-assistance.html
Texas	https://www.yourtexasbenefits.com/Screener/FindanOffice
Utah	https://jobs.utah.gov/jsp/officesearch/#/map
Vermont	https://info.healthconnect.vermont.gov/how-apply
Virginia	https://www.dss.virginia.gov/localagency/index.cgi
Washington	https://www.dshs.wa.gov/ALTSA/resources
West Virginia	https://dhhr.wv.gov/bms/Pages/Field-Offices.aspx
Wisconsin	https://www.dhs.wisconsin.gov/forwardhealth/imagency/index.htm
Wyoming	https://health.wyo.gov/healthcarefin/apply/

## Limitations

Our dataset may not capture all Medicaid offices if state government websites were not accurate or comprehensive, and should only be considered current as of late 2023 (August to December 2023). Federally qualified health centers (FQHC); community health centers; hospitals; rehabilitation and skilled nursing facilities; county and/or local health or public health departments; other state, county, and/or local government agencies (e.g., area agencies on aging); and/or other community organizations may offer Medicaid enrollment assistance; however, these were not included as they typically only assist with applications and do not have state Medicaid caseworkers on-site. Similarly, individuals and/or organizations serving as enrollment assistors or insurance navigators were not included as office locations. Future research could add these other sources of Medicaid enrollment assistance and track changes in availability of enrollment support over time, enabling more robust analysis of how the physical accessibility of enrollment assistance may affect enrollment, retention, and other programmatic outcomes. Text parsing and/or AI tools could help automate such data collection in the future, reducing the burdens of data collection. We did not review all county social service agency websites in the United States, only those county social service agency websites explicitly linked to by a state Medicaid agency website for details on office locations were reviewed (i.e., no locations listed on state agency website, only a link provided). For county social service agency websites reviewed, if they did not describe what programs and services were offered at each of their locations, we included all of them; otherwise, only offices serving Medicaid were included. State and/or county mobile units and other temporary locations were not included.

## Ethics statement

The authors have read and followed the ethical requirements for publication in *Data in Brief* and confirm that the current work does not involve human subjects, animal experiments, or any data collected from social media platforms.

## CRediT authorship contribution statement

**Paul R. Shafer:** Conceptualization, Methodology, Data curation, Formal analysis, Writing – original draft, Supervision. **Maxwell Palmer:** Methodology, Formal analysis, Writing – review & editing. **Ahyoung Cho:** Methodology, Data curation, Writing – review & editing. **Mara Lynch:** Methodology, Data curation, Writing – review & editing. **Pierce Louis:** Methodology, Data curation, Writing – review & editing. **Alexandra Skinner:** Methodology, Data curation, Writing – review & editing.

## Data Availability

Geocoded Medicaid office locations in the United States (Original data) (Harvard Dataverse).
